# Synthesis, Fluorescence Properties, and Antiproliferative Potential of Several 3-Oxo-3*H*-benzo[*f*]chromene-2-carboxylic Acid Derivatives

**DOI:** 10.3390/molecules201018565

**Published:** 2015-10-13

**Authors:** Xiao-Bo Fu, Xian-Fu Wang, Jia-Nian Chen, De-Wen Wu, Ting Li, Xing-Can Shen, Jiang-Ke Qin

**Affiliations:** State Key Laboratory Cultivation Base for the Chemistry and Molecular Engineering of Medicinal Resources, School of Chemistry & Pharmacy, Guangxi Normal University, Yucai Road 15, Guilin 541004, China; E-Mails: txzxconstance@163.com (X.-B.F.); wxf3982296@sina.com (X.-F.W.); wdw3982296@sina.com (D.-W.W.); litingfendou@163.com (T.L.); xcshen@mailbox.gxnu.edu.cn (X.-C.S.); jiangkeq@sina.com (J.-K.Q.)

**Keywords:** 3-oxo-3*H*-benzo[*f*]chromene-2-carboxylic acid derivatives, proliferation inhibitory activity, photophysical property, antitumor, biological imaging

## Abstract

In this study, two series of 3-oxo-3*H*-benzo[*f*]chromene-2-carboxylic acid derivatives (compounds **5a**–**i** and **6a**–**g**) were synthesized. Their *in vitro* proliferation inhibitory activities against the A549 and NCI-H460 human non-small cell lung cancer (NSCLC) cell lines were evaluated. Their photophysical properties were measured. Among these target compounds, **5e** exhibited the strongest antiproliferative activity by inducing apoptosis, arresting cell cycle, and elevating intracellular reactive oxygen species (ROS) level, suggesting that it may be a potent antitumor agent. In addition, compound **6g** with very low cytotoxicity, demonstrated excellent fluorescence properties, which could be used as an effective fluorescence probe for biological imaging.

## 1. Introduction

Coumarin (1,2-benzopyrone or 2*H*-1-benzopyran-2-one) and its derivatives, best-known as oxygen-containing heterocyclic compounds, are widely distributed in Nature and many exhibit diverse biological and interesting pharmacological activities. The numerous therapeutic applications of coumarin compounds include anti-coagulant effects [1], antitumor therapy [2,3,4,5], anti-HIV treatment [6,7], central nervous system stimulants and protective agents [8,9], antibacterial and anti-inflammatory drugs [10,11,12]. It has been found that the coumarin nucleus plays an important role as a valuable molecular template for the development of different structural analogues with improved pharmacological profiles. The different substituent groups connected with the coumarin nucleus strongly influence the biological activity of the resulting derivatives [13]. More importantly, the excellent fluorescent properties of coumarin compounds offer additional development value and they are used in many areas, such as fluorescent brighteners, fluorescent probes to monitor complex biological events, electroluminescent devices, photochemotherapy, *etc.* [14,15,16].

**Scheme 1:**
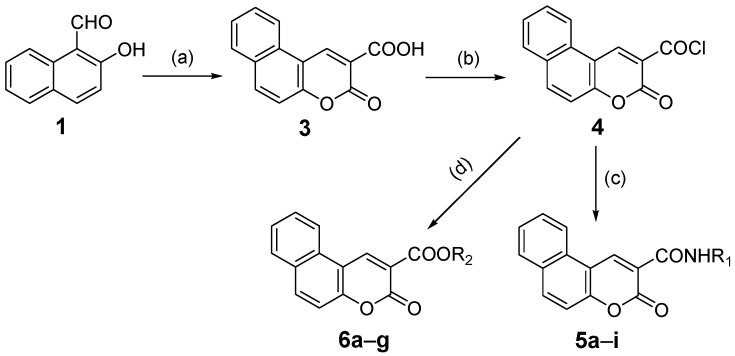
Synthetic route to the target compounds 3-oxo-3*H*-benzo[*f*]chromene-2-carboxylic acid derivatives **5a**–**i** and **6a**–**g**. *Reagents and conditions:* (**a**) Meldrum’s acid (**2**), ethanol, pyridine, 80 °C, 4 h; (**b**) SOCl_2_, 1,2-dichloroethane, 60–70 °C, 4 h; (**c**) R_1_-NH_2_, triethylamine, CH_2_Cl_2_, room temperature (r.t.), 10–30 min; (**d**) R_2_-OH, triethylamine, CH_2_Cl_2_, r.t., 10–30 min.

Recently we focused on anti-tumor activity screening and fluorescent performance of some coumarin-based compounds. In this report, two series of 3-oxo-3*H*-benzo[*f*]chromene-2-carboxylic acid derivatives including its amide (compounds **5a**–**i**) and ester (compounds **6a**–**g**) were efficiently synthesized (Scheme 1). Their biological activity and photophysical properties were evaluated. It was found that compound **5e** exhibited the strongest *in vitro* proliferation inhibitory activity against the A549 and NCI-H460 cell lines, suggesting that it may be a potent antitumor agent. In addition, compound **6g** with very low cytotoxicity demonstrated excellent fluorescence properties, which could be exploited for biological and biomedical imaging.

## 2. Results and Discussion

### 2.1. Chemistry

The synthetic route to the target 3-oxo-3*H*-benzo[*f*]chromene-2-carboxylic acid derivatives **5a**–**i** and **6a**–**g** is depicted in Scheme 1. Based on the method reported previously [17], we successfully prepared the key intermediate 3-oxo-3*H*-benzo[*f*]chromene-2-carboxylic acid (**3**) via Knoevenagel condensation of 2-hydroxy-1-naphthaldehyde (**1**) with Meldrum’s acid (**2**). For the first step, cheap ethanol was selected as the solvent and pyridine as a catalyst. It was noted that an excess of Meldrum’s acid should be avoided because it could react with **3** [18]. Then 3-oxo-3*H*-benzo[*f*]chromene-2-carbonyl chloride (**4**) was obtained by chlorination in the presence of thionyl chloride. Compound **4** is easily hydrolyzed and converted back into the previous carboxylic acid **3** if it is exposed to the air for a long time, therefore **4** was used directly in the next step without further purification. Finally, different amines and alcohols were reacted with **4** to afford the target compounds **5a**–**i** and **6a**–**g** (Scheme 1). This method is convenient, efficient and can be used for constructing coumarin-based compound libraries; moreover, the byproducts are easy to remove, and the intermediate **3** is obtained in high yield and purity.

**Scheme 2:**
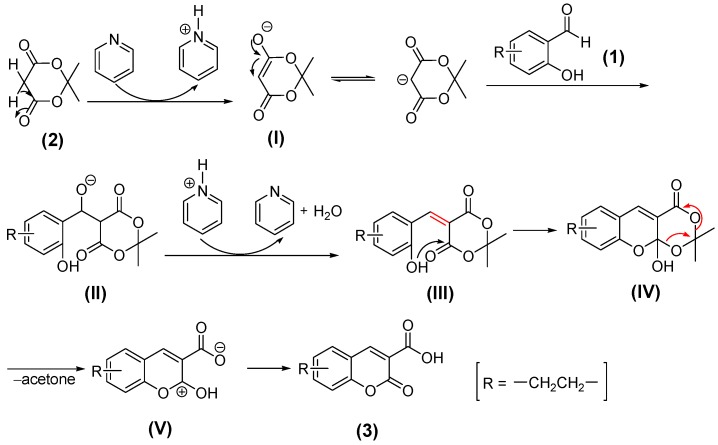
Proposed mechanism of the pyridine-catalyzed Knoevenagel condensation for synthesizing 3-oxo-3*H*-benzo[*f*]chromene-2-carboxylic acid (**3**).

After the target compounds were successfully prepared, the reaction mechanism for synthesizing the key intermediate 3-oxo-3*H*-benzo[*f*]chromene-2-carboxylic acid (**3**) was examined. As shown in Scheme 2, Meldrum’s acid (**2**), namely 2,2-dimethyl-1,3-dioxane-4,6-dione, is easily enolized and usually used as a good nucleophile in many reactions when malonate ester is ineffective [19]. In the presence of pyridine, Meldrum’s acid (**2**) is deprotonated to afford its enolate **I** which can be converted to the corresponding ketone form. Then the produced carbanion attacks the 2-hydroxy-1-naphthaldehyde substrate **1**, and the intermediate **II** is obtained. However, **II** is prone to protonate, then lose a water molecule, and the olefine ketone **III** is produced [18,20]; meanwhile, the catalyst pyridine restores its initial state. Subsequently, an intramolecular nucleophilic addition occurs because the carbonyl carbon of **III** is susceptible to attack by the hydroxyl group, which gives the hemiacetal **IV**. The strain of the rightmost lactone ring combined with instability of the hemiacetal structure forces the loss of an acetone molecule, and the intermediate **V** is thus obtained [19,21]. However, because the hydroxyl group is directly linked with a carbocation, **V** is also unstable and easily loses a proton, which is captured by the carboxylate anion to form the carboxyl group and 3-oxo-3*H*-benzo[*f*]chromene-2-carboxylic acid (**3**) is obtained. Of course, with regard to the conversion from **V** to **3**, there may be another path where the proton of the carboxyl group comes from a water molecule which was generated previously, rather than the hydroxyl group of **V**. From the above analysis, many proton-binding agents can be used as catalysts in the Knoevenagel condensation. Besides pyridine, maybe other bases, such as piperidine, piperidinium acetate, piperazine, and ammonium acetate are also appropriate [20,22].

### 2.2. In Vitro Biological Activity

#### 2.2.1. Antiproliferative Activities of the Target Compounds against A549 and NCI-H460 Cells *in Vitro*

**Figure 1:**
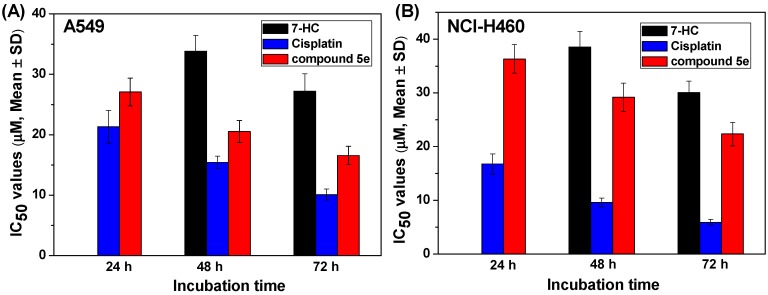
Antiproliferative effects of compound **5e** on NSCLC cell lines. (**A**) A549 and (**B**) NCI-H460 cells were treated with 7-HC, cisplatin, and **5e** for the indicated time (24, 48, and 72 h), respectively. After treatment, cell viability was measured by MTT assay, then inhibition rate and IC_50_ values were obtained. In the figure, the antiproliferative activity data of 7-HC after the incubation with A549 and NCI-H460 cell lines for 24 h were omitted because the IC_50_ values were over 60 μM under this condition.

The *in vitro* proliferation inhibitory activity of the prepared compounds **5a**–**i** and **6a**–**g** was evaluated against two NSCLC cell lines including A549 and NCI-H460; 7-hydroxycoumarin (7-HC) and cisplatin were selected as dual positive reference drugs. The NSCLC cell lines were treated with the target compounds (final concentrations: 0, 5, 10, 20, 30, 40, 60 μM), and viable cells were measured by an MTT (3-(4,5-dimethylthiahiazol-2-y1)-2,5-diphenyltetrazolium bromide) assay. As shown in Table 1, most of the target compounds exhibited very low antiproliferative activities; especially, the IC_50_ values (IC_50_ represented the concentration at which cell growth was inhibited by 50%) of 3-oxo-3*H*-benzo[*f*]chromene-2-carboxylic acid esters **6a**–**g** were over 60 μM. To our delight, among the synthesized compounds, **5e** displayed the best activities with IC_50_ values of 20.53 ± 1.84 for A549 and 29.19 ± 2.61 for NCI-H460 cells, respectively. After the co-incubation of A549 cells with 10, 20, and 30 μM compounds **5e** for 48 h, the inhibition rates were 22.55% ± 1.97%, 52.59% ± 2.79% and 63.72% ± 3.09%, respectively. The decline of IC_50_ values with the prolonged incubation time is shown in Figure 1. At the same concentration, the proliferation inhibitory activity of **5e** was superior to 7-HC, even though neither of them are comparable with cisplatin which has been extensively used to treat patients with NSCLC within the last several decades [23,24,25]. The above results suggest that compound **5e** can induce a dose- and time-dependent proliferation inhibition of A549 and NCI-H460 cells.

**Table 1:** *In vitro* antiproliferative activities of 3-oxo-3*H*-benzo[*f*]chromene-2-carboxylic acid derivatives against A549 and NCI-H460 cell lines. 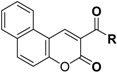

Compound	R	IC_50_ ± SD (μM) ^a,b^	
		A549	NCI-H460
**5a**	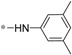	>60	46.41 ± 4.22
**5b**	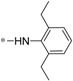	>60	>60
**5c**	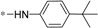	>60	>60
**5d**	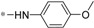	38.71 ± 3.62	44.39 ± 2.98
**5e**	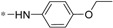	20.53 ± 1.84	29.19 ± 2.61
**5f**	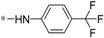	29.72 ± 3.85	>60
**5g**	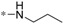	>60	>60
**5h**	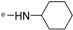	>60	>60
**5i**	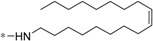	>60	>60
**6a**	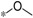	>60	>60
**6b**	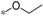	>60	>60
**6c**	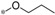	>60	>60
**6d**	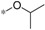	>60	>60
**6e**	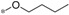	>60	>60
**6f**	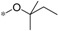	>60	>60
**6g**	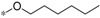	>60	>60
7-HC	—	33.82 ± 2.62	38.55 ± 2.91
Cisplatin	—	15.42 ± 1.06	9.61 ± 0.83

^a^ A549 and NCI-H460 cells were treated with the target compounds for 48 h. Results are expressed as means ± SD (standard deviation) of four independent experiments; ^b^ Compounds with IC_50_ values >60 μM are considered to be inactive.

#### 2.2.2. Apoptosis Induced by Compound **5e**

**Figure 2:**
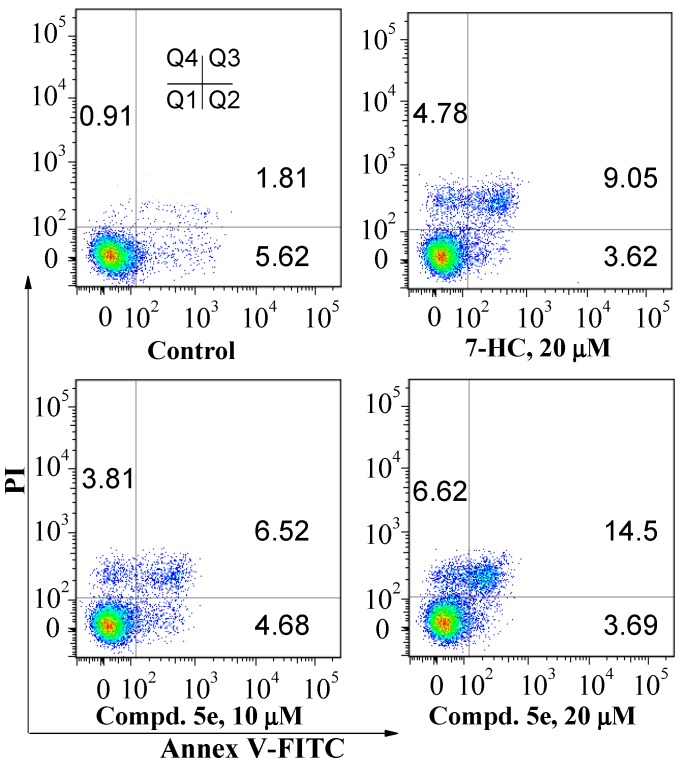
Effects of compound **5e** on the induction of apoptosis in A549 cells after the co-incubation for 72 h. The population of apoptotic cells was determined by flow cytometry. The experiment was repeated thrice. Here only representative flow cytometric graphs are shown.

To investigate whether the proliferation inhibitory activity of compound **5e** was associated with induced apoptosis, Annexin V-FITC/propidium iodide (Annexin V/PI) dual staining method was used. As shown in Table 2 and Figure 2, after A549 cells were treated with compound **5e** for 48 h, the population of apoptotic cells (early plus late apoptotic cells, Q2 + Q3 quadrants) increased significantly. Compared with 7-HC, **5e** had more potent capability to induce apoptosis at the same concentration (20 µM). If the incubation time was extended to 72 h, the population of apoptotic cells was increased from 7.22% ± 0.52% for the control group to 10.9% ± 2.1% at 10 µM, then to 19.1% ± 1.4% at 20 μM for **5e** (Table 2). Therefore, compound **5e** can induce apoptosis of A549 cells in a concentration- and time-dependent manner; induced apoptosis is responsible for the antiproliferative activity of compound **5e** against A549 cells.

**Table 2:** Quantitative apoptosis assay of A549 using Annexin-V/PI dual staining method ^a^.

Group	Conc. ^b^ (µM)	48 h Incubation			72 h Incubation		
		Viable Cells (Q1, %)	Apoptotic Cells (Q2 + Q3, %)	Necrotic Cells (Q4, %)	Q1 (%)	Q2 + Q3 (%)	Q4 (%)
Control	0	94.7 ± 2.6	4.72 ± 0.41	0.62 ± 0.19	91.8 ± 3.3	7.22 ± 0.52	0.97 ± 0.13
7-HC	20	85.7 ± 2.8	11.3 ± 1.4 **	3.04 ± 0.29	81.1 ± 2.4	14.2 ± 1.9 *	4.71 ± 0.49
Compd. **5e**	10	91.4 ± 3.1	6.51 ± 1.31	2.04 ± 0.31	85.3 ± 2.9	10.9 ± 2.1	3.77 ± 0.31
Compd. **5e**	20	80.7 ± 2.5	13.9 ± 2.2 **	5.41 ± 0.62	73.9 ± 2.7	19.1 ± 1.4 **	6.98 ± 0.42

^a^ A549 cells were treated with the indicated concentrations of compound **5e** for 48 and 72 h, respectively; subsequently the cells were stained with Annexin V-FITC and PI. The percentage of viable cells, apoptotic cells and necrotic cells is expressed as the means ± SD from three independent experiments; ^b^ Conc. is the abbreviation of concentration; * *p* < 0.05; ** *p* < 0.01 *vs.* the percentage of apoptotic cells of the control.

#### 2.2.3. Cell Cycle Analysis

**Figure 3:**
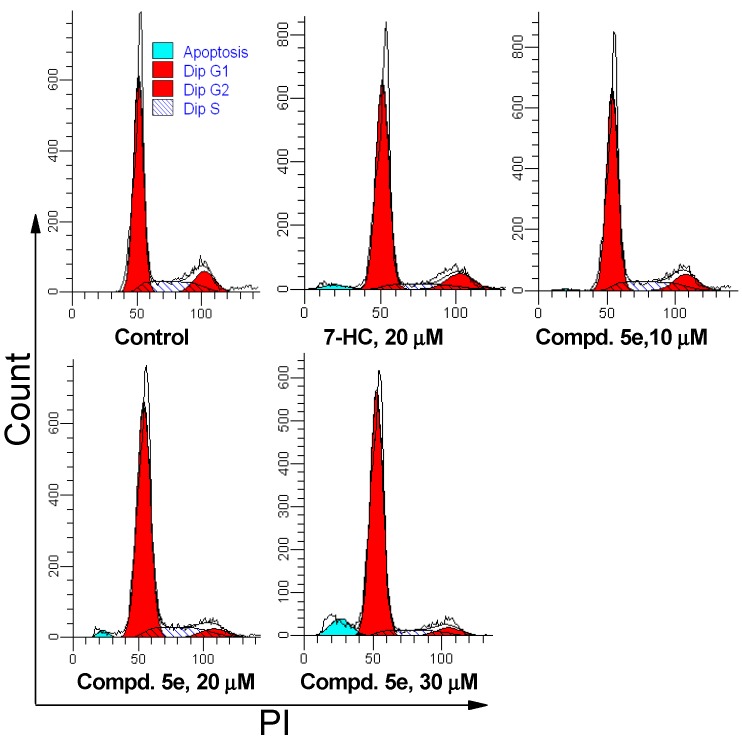
Effects of compound **5e** on A549 cell cycle progression. A549 cells were treated with **5e** for 72 h and analyzed by flow cytometry. The experiment was repeated thrice. Here only representative flow cytometric graphs are shown.

Cell cycle distribution was examined to determine whether compound **5e** inhibited the proliferation of A549 cells through cell cycle arrest. The cells were co-incubated with **5e** and 7-HC for 48 and 72 h, respectively. Compound **5e** treatment increased the population of cells in the G0/G1 phase in a concentration- and time-dependent manner. This was accompanied by a decrease in the population of cells in S and G2/M phases, compared with the control. As shown in Table 3 and Figure 3, the population of A549 cells in the G0/G1 phase was increased from 70.71% ± 2.79% (control) to 80.52% ± 2.29% at 20 μM, then to 85.51% ± 2.03% at 30 μM for **5e**; meanwhile, apoptotic cell rate was elevated with increased dose, which was consistent with the above results of induced apoptosis. These data indicate that, besides induced apoptosis, cell cycle arrest in the G0/G1 stage is also an important factor by which compound **5e** exerts its inhibitory effects on A549 cells.

**Table 3:** Effects of compound **5e** on cell cycle progression in A549 cells ^a^.

Group	Conc. (µM)	G0/G1 Phase (%)	S Phase (%)	G2/M Phase (%)
Control	0	70.71 ± 2.79	16.79 ± 1.37	12.50 ± 1.41
7-HC	20	79.83 ± 2.39 *	8.38 ± 0.44	11.79 ± 1.69
Compd. **5e**	10	72.62 ± 2.67	15.77 ± 0.84	11.60 ± 1.31
Compd. **5e**	20	80.52 ± 2.29 *	13.14 ± 1.01	6.31 ± 0.62
Compd. **5e**	30	85.51 ± 2.03 *	8.82 ± 0.91	5.67 ± 0.43

^a^ A549 cells were treated with compound **5e** or 7-HC for 72 h, then DNA content was analyzed through PI staining. Flow cytometry data were analyzed using the ModFit software. The results are presented as the means ± SD of three independent experiments; * *p* < 0.05 *vs.* the percentage of cells in G0/G1 phase of the control.

#### 2.2.4. Induced ROS Generation by Compound **5e**

**Figure 4:**
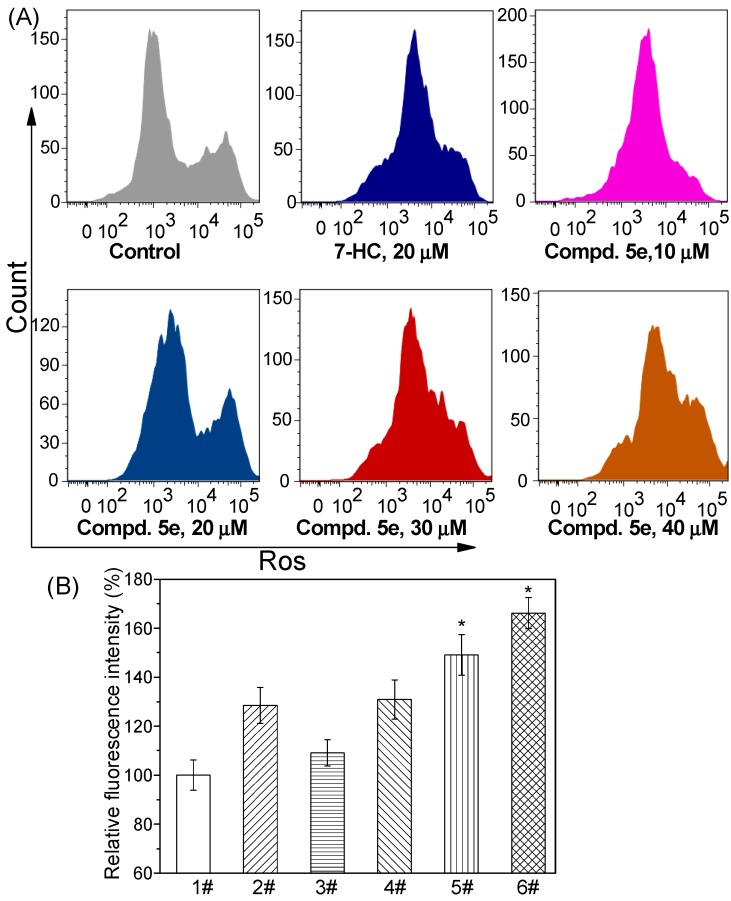
Effects of compound **5e** on the intracellular ROS level in A549 cells. (**A**) ROS level analysis using flow cytometry; (**B**) Quantitative analysis of relative fluorescence intensity (% of control) using a luminescence spectrometer. A549 cells were treated with **5e** for 48 h and stained with 2′,7′-dichlorodihydrofluorescein diacetate (DCFH-DA) before monitoring ROS level. Notes of Figure 4B, 1#: Control; 2#: 7-HC (20 µM); 3#: **5e** (10 µM); 4#: **5e** (20 µM); 5#: **5e** (30 µM); 6#: **5e** (40 µM). Each bar represents the mean ± SD of three independent experiments; * *p* < 0.05 *vs.* control.

The intracellular ROS levels were measured before and after treatment with compound **5e**. As shown in Figure 4A, exposure to over 20 μM of **5e** caused a marked increase in fluorescence intensity of 2′,7′-dichlorofluorescein (DCF), which indicated the production of ROS in the mitochondria of A549 cells. Intracellular fluorescence intensity was further quantitatively analyzed using a luminescence spectrometer. The mean fluorescence intensities increased by 30.9%, 49.1%, and 66.1% after treatment with 20, 30, and 40 μM compound **5e**, respectively. Similar results were obtained for 7-HC. The relative fluorescence intensity (% of control) was shown in Figure 4B after the cells were co-incubated with **5e** for 48 h. ROS overproduction is implicated in mediation of apoptosis and has been described as an early event [26]. Elevated intracellular ROS levels indicate that the balance between ROS generation and elimination is disrupted, which eventually results in cell apoptosis. Therefore, mitochondrial ROS overproduction and malfunction are responsible for compound **5e**-induced apoptosis.

### 2.3. Absorption Spectra, Fluorescence Spectra, and Living Cell Imaging

**Figure 5:**
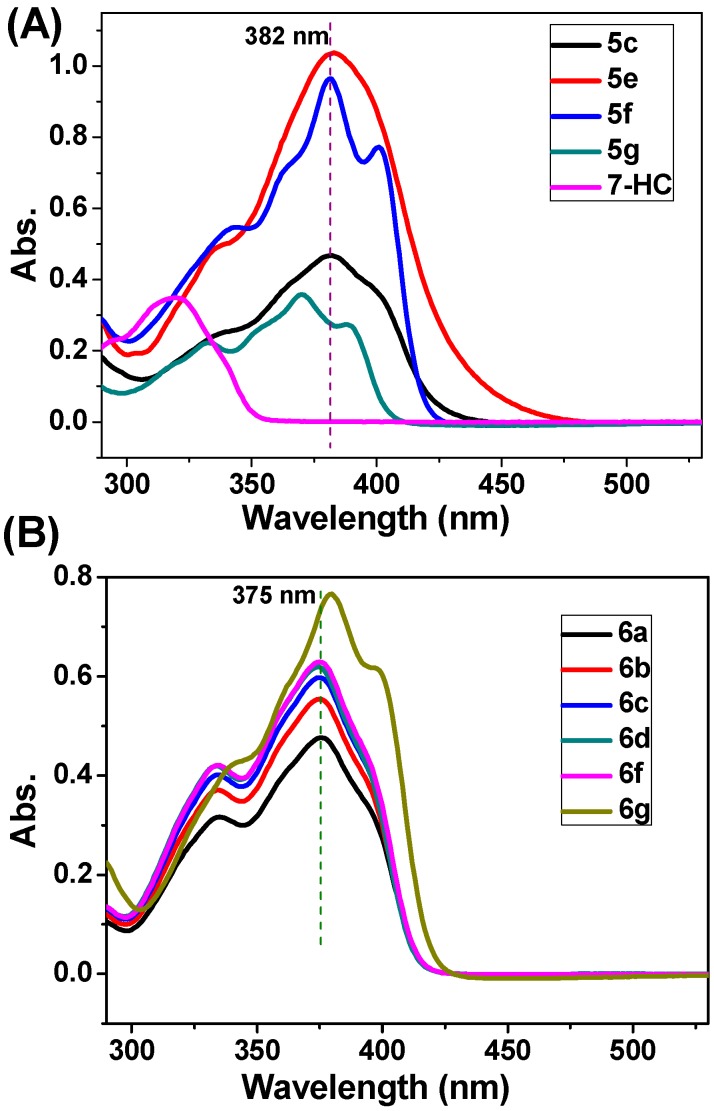
The absorption spectra of several representative compounds. (**A**) Compounds **5c**, **5e**, **5f**, **5g**, 7-HC; (**B**) Compounds **6a**, **6b**, **6c**, **6d**, **6f**, **6g**. The above compounds (final concentration: 50 μM**)** were dissolved in 1,2-dichloroethane.

**Figure 6:**
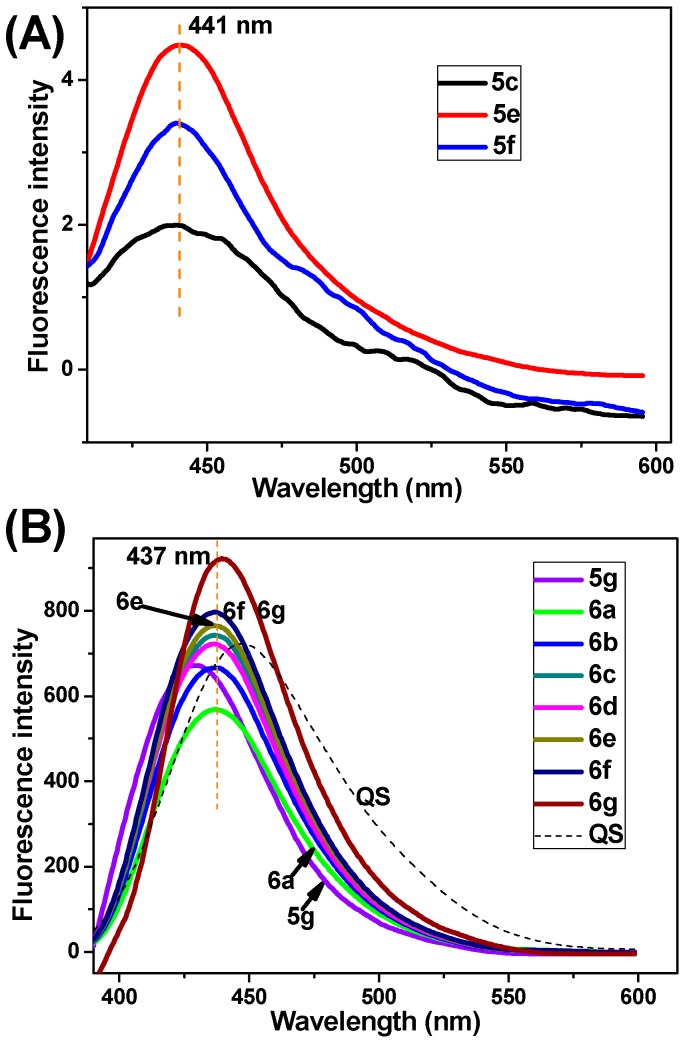
The emission spectra of several representative compounds. (**A**) Compounds **5c**, **5e**, **5f**; (**B**) Compounds **5g**, **6a**, **6b**, **6c**, **6d**, **6e**, **6f**, **6g**. The above compounds (final concentration: 5 μM**)** were dissolved in 1,2-dichloroethane (λ_ex_ = 360 nm).

Fluorescence bioimaging provides a facile and less cell-damaging means of visualizing analytes of biological interest in living cells. From the viewpoint of the potential application of the synthesized 3-oxo-3*H*-benzo[*f*]chromene-2-carboxylic acid derivatives in biological imaging, we investigated these compounds’ photophysical performance. Firstly, the ultraviolet-visible (UV-Vis) absorption spectra of several representative compounds were recorded in 1,2-dichloroethane solution (final concentration: 50 μM). As shown in Figure 5, these compounds have a nearly identical absorption band ranging from 360 to 410 nm. The fluorescence spectra in the excitation of 360 nm were recorded at the concentration of 5 μM (Figure 6). The compounds **5g** and **6a**–**g** produce a strong blue emission band in the range of 430–445 nm, in comparison with 7-HC which has very weak fluorescence emission at 396 nm. At the same concentration (e.g., 5 μM), the fluorescence intensities of 3-oxo-3*H*-benzo[*f*]chromene-2-carboxylic acid esters **6a**–**g** are sequentially elevated with the increase in the chain length of the terminal alkyl groups (Figure 6B). Their fluorescence quantum yields (Φ_f_) in the solution state were determined using the standard procedures, with quinine sulphate (QS) dissolved in 0.1 M sulphuric acid as a reference standard (Φ_f_ = 0.54) [27]. The UV-Vis and fluorescence data including the absorption (λ_abs_) and fluorescence (λ_em_) maximal values, Stokes shift (ν_abs_ − ν_em_), and Φ_f_ are summarized in Table 4. Compared with 7-HC, a remarkable red shift phenomenon in absorption and emission spectra of compounds **5g** and **6a**–**g** was observed. The above eight compounds display good Φ_f_ in the range of 0.44–0.66, which is superior to compounds **5a**–**f**, **5h**, **5i**, and 7-HC with very low Φ_f_ (<0.1). Compound **6g** has the highest Φ_f_ of 0.66. It is quite interesting that, as far as 3-oxo-3*H*-benzo[*f*]chromene-2-carboxylic acid esters are concerned, the Φ_f_ regularly increase with the prolongation of aliphatic chain, which is coincide with the changing trend of fluorescence intensity. Moreover, the Φ_f_ value of *n*-propyl ester is higher than *i*-propyl ester, suggesting that linear chain alkyl group may lead to the increase of Φ_f_, compared with branched chain alkyl group if their carbon atoms in the alcoholic portion are equal.

**Table 4:** Optical data of several representative compounds.

Entry	Compound	λ_abs,_ nm ^a^	λ_em,_ nm ^a^	Stokes Shift ^b^		Φ_f_
1	**5c**	383	442	3485 cm^−1^	59 nm	<0.1
2	**5e**	383	441	3433 cm^−1^	58 nm	<0.1
3	**5f**	381	440	3519 cm^−1^	59 nm	<0.1
4	**5g**	370	434	3986 cm^−1^	64 nm	0.49
5	**6a**	375	437	3783 cm^−1^	62 nm	0.44
6	**6b**	375	437	3783 cm^−1^	62 nm	0.51
7	**6c**	375	437	3783 cm^−1^	62 nm	0.58
8	**6d**	375	437	3783 cm^−1^	62 nm	0.52
9	**6e**	375	437	3783 cm^−1^	62 nm	0.60
10	**6f**	375	437	3783 cm^−1^	62 nm	0.62
11	**6g**	380	441	3640 cm^−1^	61 nm	0.66
12	7-HC	319	396	6095 cm^−1^	71 nm	<0.1
13	QS ^c^	348	445	6264 cm^−1^	97 nm	0.54

^a^ Here refers to the λ_abs_ and λ_em_ values where the UV-Vis absorption and fluorescence intensity are maximum, respectively; ^b^ Stokes shifts are listed in units of both inverse centimeters and nanometers; ^c^ QS dissolved in 0.1 M sulphuric acid is selected as a reference standard to calculate the Φ_f_ of the synthesized target compounds.

**Figure 7:**
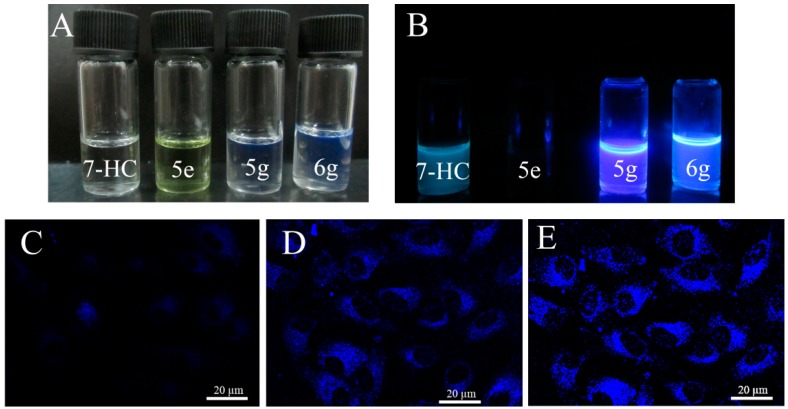
Fluorescence and living cell imaging of representative compounds. (**A**) Color responses to visible light; (**B**) Fluorescence responses after excitation at 365 nm using an UV lamp; (**C**–**E**) CLSM images after A549 cells were treated with 20 μM of 7-HC, **5g**, and **6g** for 30 min, respectively.

After this, the application of the synthesized target compounds for living cell imaging was investigated. 7-HC, **5e**, **5g**, and **6g** were selected as representative examples. Compounds **5g** and **6g**, dissolved in 1,2-dichloroethane and exposed to visible light, exhibited conspicuous blue fluorescence. However, almost no fluorescence was observed for 7-HC and **5e** under the same conditions (Figure 7A). The former two emitted very strong fluorescence once they were exposed to ultraviolet light for a few seconds (Figure 7B). Living cells images were monitored with a confocal laser scanning microscope (CLSM) after treatment with the above compounds (Figure 7C–E). Emissions of 430–450 nm were collected when a laser at 360 nm was used as excitation. The cytoplasmic was selectively dyed (blue) by **5g** and **6g**; the nucleus was undyed (clear). Moreover, at the same concentration of 20 μM, intracellular fluorescence intensities: **6g** > **5g** > 7-HC. The cells stained with 7-HC released very weak fluorescence maybe due to its very low Φ_f_, which was consistent with the previous report [28]. Photophysical studies reveal that at least compounds **5g** and **6g** with very low cytotoxicity are potential fluorescent probes for biological imaging in living cells.

## 3. Experimental Section

### 3.1. Chemistry

#### 3.1.1. Material and Reagents

Proton nuclear magnetic resonance (^1^H-NMR) and ^13^C-NMR spectra were recorded in CDCl_3_ (or DMSO-*d*_6_) on a Bruker Avance 500 MHz NMR spectrometer at 500 and 125 MHz, respectively; chemical shifts were reported in parts per million using tetramethylsilane as an internal standard and were given in δ units. Uncorrected melting points were determined on an electrothermal melting point apparatus. High-resolution mass spectra (HRMS) were recorded on a New ultrafleXtreme MALDI TOF/TOF mass spectrometer (Bruker Daltonics Inc., Billerica, MA, USA). Column chromatography was performed on silica gel (300–400 mesh). Analytical TLC was carried out on E. Merck 0.25 mm silica gel 60 F-254 precoated plates with detection by UV light. Unless otherwise noted, all solvents and reagents were commercially available and used without further purification.

#### 3.1.2. Synthesis of 3-Oxo-3*H*-benzo[*f*]chromene-2-carboxylic Acid (**3**)

The synthesis of 3-oxo-3*H*-benzo[*f*]chromene-2-carboxylic acid (**3**) was described elsewhere [17], but is summarized here for convenience. Briefly, ethanol (85 mL), Meldrum’s acid (6.5 g, 45 mmol), 2-hydroxy-1-naphthaldehyde (7.75 g, 45 mmol), and a catalytic amount of pyridine (0.5 mL) were in sequence added into a 100 mL flask. The mixture was stirred at 80 °C for 4 h. After cooling to room temperature, the mixture was poured into an ice-water bath and recrystallized. The crude was washed with ethanol repeatedly and dried in vacuo to afford the desired product 3-oxo-3*H*-benzo[*f*]chromene-2-carboxylic acid (**3**) as a yellow solid (Yield: 93.1%). M.p.: 235.1–236.5 °C (Lit. 234–236 °C [29]). ^1^H-NMR (DMSO-*d*_6_): δ 9.34 (s, 1H), 8.57 (d, *J* = 8.4 Hz, 1H), 8.29 (d, *J* = 9.0 Hz, 1H), 8.06 (d, *J* = 8.0 Hz, 1H), 7.78–7.73 (m, 1H), 7.64 (t, *J* = 7.2 Hz, 1H), 7.57 (d, *J* = 9.0 Hz, 1H). ^13^C-NMR (DMSO-*d*_6_): δ 164.78, 157.24, 155.49, 144.18, 136.30, 130.27, 129.50, 129.45, 126.88, 122.77, 117.68, 116.94, 112.55. ESI-HRMS (*m*/*z*): calcd. for C_14_H_8_O_4_ [M − H]^−^: 239.03443; found: 239.03429.

#### 3.1.3. General Procedures for the Preparation of 3-Oxo-3*H*-benzo[*f*]chromene-2-amides **5a**–**i** and 3-Oxo-3*H*-benzo[*f*]chromene-2-carboxylic Acid Esters **6a**–**g**

First of all, 3-oxo-3*H*-benzo[*f*]chromene-2-carbonyl chloride (**4**) was synthesized by the reaction of 3-oxo-3*H*-benzo[*f*]chromene-2-carboxylic acid (**3**, 2.40 g, 10 mmol) with thionyl chloride (2.2 mL, 30 mmol) in dry 1,2-dichloroethane (20 mL) at 60–70 °C for 4 h. After the removal of excess unreacted thionyl chloride under reduced pressure, the intermediate **4** was obtained and used directly in the next step without further purification. This intermediate **4** (1.29 g, 5 mmol) dissolved in dry dichloromethane (15 mL) was dropwise added to a mixture of a primary amine (6 mmol) and a catalytic amount of triethylamine (0.1 mL). The reaction mixture was stirred at room temperature for 10–30 min before the solvent was removed. The crude was purified by flash silica gel column chromatography to afford the final product **5a**–**i**. Compounds **6a**–**g** were also synthesized using the similar procedures, the only difference being that the primary amines described above were replaced by different aliphatic alcohols.

*N-(3,5-Dimethylphenyl)-3-oxo-3H-benzo[f]chromene-2-carboxamide* (**5a**). Yield: 74.4%; m.p.: 277.1–279.2 °C. ^1^H-NMR (CDCl_3_): δ 9.82 (s, 1H), 8.50 (d, *J* = 8.3 Hz, 1H), 8.18 (d, *J* = 9.1 Hz, 1H), 7.99 (d, *J* = 7.9 Hz, 1H), 7.82 (t, *J* = 7.7 Hz, 1H), 7.69 (t, *J* = 7.6 Hz, 1H), 7.58 (d, *J* = 8.9 Hz, 1H), 7.45 (s, 2H), 6.85 (s, 1H), 2.38 (s, 6H). ^13^C-NMR (CDCl_3_): δ 161.89, 159.64, 155.04, 144.35, 138.79, 137.59, 136.17, 130.46, 129.54, 129.36, 129.21, 126.91, 126.59, 121.98, 118.32, 117.19, 116.34, 113.54, 21.45. ESI-HRMS (*m*/*z*): calcd. for C_22_H_17_NO_3_ [M + Na]^+^: 366.11061; found: 366.10929.

*N-(2,6-Diethylphenyl)-3-oxo-3H-benzo[f]chromene-2-carboxamide* (**5b**). Yield: 35.4%; m.p.: 190.3–193.5 °C. ^1^H-NMR (CDCl_3_): δ 9.82 (s, 1H), 8.50 (d, *J* = 8.3 Hz, 1H), 8.18 (d, *J* = 9.0 Hz, 1H), 7.99 (d, *J* = 8.0 Hz, 1H), 7.82–7.78 (m, 1H), 7.68 (m, 1H), 7.59 (d, *J* = 9.1 Hz, 1H), 7.30 (dd, *J* = 12.4, 5.3 Hz, 1H), 7.22 (d, *J* = 7.6 Hz, 2H), 2.71 (q, *J* = 7.6 Hz, 4H), 1.26 (t, *J* = 7.6 Hz, 6H). ^13^C-NMR (CDCl_3_): δ 162.10, 160.85, 155.15, 144.79, 141.22, 136.17, 132.66, 130.45, 129.56, 129.28, 129.17, 128.01, 126.90, 126.42, 122.16, 116.89, 116.36, 113.47, 25.15, 14.52. ESI-HRMS (*m*/*z*): calcd. for C_24_H_21_NO_3_ [M + Na]^+^: 394.14191; found: 394.14231.

*N-(4-(Tert-butyl)phenyl)-3-oxo-3H-benzo[f]chromene-2-carboxamide* (**5c**). Yield: 22.7%; m.p.: 180.2–181.9 °C. ^1^H-NMR (CDCl_3_): δ 9.79 (s, 1H), 8.49 (d, *J* = 8.4 Hz, 1H), 8.16 (d, *J* = 9.0 Hz, 1H), 7.97 (d, *J* = 8.1 Hz, 1H), 7.84–7.77 (m, 1H), 7.69–7.63 (m, 1H), 7.56 (d, *J* = 9.0 Hz, 1H), 7.48–7.43 (m, 2H), 7.25–7.19 (m, 1H), 6.71–6.64 (m, 1H), 1.38 (s, 9H). ^13^C-NMR (CDCl_3_): δ 161.87, 159.58, 154.98, 147.80, 144.26, 143.70, 141.48, 136.09, 135.18, 130.43, 129.24, 126.89, 125.99, 122.02, 120.30, 117.14, 116.32, 114.98, 113.51, 34.49, 33.93, 31.40. ESI-HRMS (*m*/*z*): calcd. for C_24_H_21_NO_3_ [M + Na]^+^: 394.14191; found: 394.14090.

*N-(4-Methoxyphenyl)-3-oxo-3H-benzo[f]chromene-2-carboxamide* (**5d**). Yield: 61.2%; m.p.: 259.3–261.8 °C (Lit. 259–261 °C [30]). ^1^H-NMR (CDCl_3_): δ 9.82 (s, 1H), 8.51 (d, *J* = 8.4 Hz, 1H), 8.18 (d, *J* = 9.0 Hz, 1H), 7.99 (d, *J* = 8.1 Hz, 1H), 7.82 (t, *J* = 7.7 Hz, 1H), 7.73 (d, *J* = 8.9 Hz, 2H), 7.68 (t, *J* = 7.5 Hz, 1H), 7.58 (d, *J* = 9.0 Hz, 1H), 6.97 (d, *J* = 8.9 Hz, 2H), 3.86 (s, 3H). ^13^C-NMR (CDCl_3_): δ 161.93, 156.70, 154.98, 144.19, 136.08, 131.01, 130.47, 129.53, 129.30, 129.18, 126.89, 122.09, 122.04, 117.18, 116.34, 114.23, 113.56, 55.51. ESI-HRMS (*m*/*z*): calcd. for C_21_H_15_NO_4_ [M + Na]^+^: 368.08988; found: 368.08763.

*N-(4-Ethoxyphenyl)-3-oxo-3H-benzo[f]chromene-2-carboxamide* (**5e**). Yield: 80.3%; m.p.: 205.1–206.6 °C. ^1^H-NMR (CDCl_3_): δ 9.82 (s, 1H), 8.51 (d, *J* = 8.4 Hz, 1H), 8.17 (d, *J* = 9.0 Hz, 1H), 7.99 (d, *J* = 8.1 Hz, 1H), 7.82 (t, *J* = 7.2 Hz, 1H), 7.77–7.62 (m, 3H), 7.58 (d, *J* = 9.0 Hz, 1H), 7.04–6.82 (m, 2H), 4.08 (d, *J* = 7.0 Hz, 2H), 1.46 (s, 3H). ^13^C-NMR (CDCl_3_): δ 161.94, 159.45, 156.10, 154.99, 144.17, 136.06, 130.91, 130.48, 129.55, 129.31, 129.19, 126.89, 122.08, 117.22, 116.36, 114.85, 113.58, 63.72, 14.88. ESI-HRMS (*m*/*z*): calcd. for C_22_H_17_NO_4_ [M + H]^+^: 360.12358; found: 360.12223.

*3-Oxo-N-(4-(trifluoromethyl)phenyl)-3H-benzo[f]chromene-2-carboxamide* (**5f**). Yield: 73.9%; m.p.: 231.3–233.9 °C. ^1^H-NMR (CDCl_3_): δ 9.81 (s, 1H), 8.49 (d, *J* = 8.3 Hz, 1H), 8.16 (d, *J* = 9.0 Hz, 1H), 7.98 (d, *J* = 8.0 Hz, 1H), 7.81 (t, *J* = 7.7 Hz, 1H), 7.67 (t, *J* = 7.6 Hz, 1H), 7.57 (d, *J* = 8.9 Hz, 1H), 7.44 (s, 2H), 7.27 (s, 1H), 6.84 (s, 1H). ^13^C-NMR (CDCl_3_): δ 161.97, 160.16, 155.29, 145.02, 140.81, 136.69, 130.50, 129.54, 129.53, 129.50, 129.30, 127.08, 126.36, 126.34, 121.98, 120.23, 116.55, 116.34, 113.50. ESI-HRMS (*m*/*z*): calcd. for C_21_H_12_F_3_NO_3_ [M − H]^−^: 382.06910; found: 382.06886.

*3-Oxo-N-propyl-3H-benzo[f]chromene-2-carboxamide* (**5g**). Yield: 88.1%; m.p.: 182.3–184.9 °C. ^1^H-NMR (CDCl_3_): δ 9.70 (s, 1H), 8.47 (d, *J* = 8.4 Hz, 1H), 8.13 (d, *J* = 9.0 Hz, 1H), 7.96 (d, *J* = 8.1 Hz, 1H), 7.79 (t, *J* = 7.5 Hz, 1H), 7.68–7.61 (m, 1H), 7.53 (d, *J* = 9.0 Hz, 1H), 3.50 (dd, *J* = 13.0, 7.0 Hz, 2H), 1.77–1.68 (m, 2H), 1.05 (t, *J* = 13.5 Hz, 3H). ^13^C-NMR (CDCl_3_): δ 161.86, 161.60, 154.83, 143.80, 135.70, 130.39, 129.56, 129.13, 126.75, 122.04, 117.08, 116.33, 113.38, 41.67, 22.74, 11.56. ESI-HRMS (*m*/*z*): calcd. for C_17_H_15_NO_3_ [M + Na]^+^: 304.09496; found: 304.09352.

*N-Cyclohexyl-3-oxo-3H-benzo[f]chromene-2-carboxamide* (**5h**). The process for the synthesis of compound **5h** was described previously [31]. Yield: 89.1%; m.p.: 178.6–180.2 °C. ^1^H-NMR (CDCl_3_): δ 9.72 (s, 1H), 8.49 (d, *J* = 8.4 Hz, 1H), 8.14 (d, *J* = 9.0 Hz, 1H), 7.97 (d, *J* = 8.1 Hz, 1H), 7.83–7.77 (m, 1H), 7.69–7.63 (m, 1H), 7.54 (d, *J* = 9.0 Hz, 1H), 4.12–4.01 (m, 1H), 2.04 (m, 2H), 1.84–1.77 (m, 2H), 1.70–1.55 (m, 2H), 1.52–1.35 (m, 2H), 1.32–1.19 (m, 2H). ^13^C-NMR (CDCl_3_): δ 161.60, 160.83, 154.81, 143.76, 135.64, 130.40, 129.58, 129.14, 129.11, 126.73, 122.08, 117.29, 116.36, 113.43, 48.61, 32.80, 29.72, 25.62, 24.70. ESI-HRMS (*m*/*z*): calcd. for C_20_H_19_NO_3_ [M + H]^+^: 322.14432; found: 322.14298.

*(Z)-N-(Octadec-9-en-1-yl)-3-oxo-3H-benzo[f]chromene-2-carboxamide* (**5i**). Yield: 60.9%; m.p.: 79.1–81.8 °C. ^1^H-NMR (CDCl_3_): δ 9.62 (s, 1H), 8.40 (d, *J* = 8.5 Hz, 1H), 8.08 (d, *J* = 9.0 Hz, 1H), 7.91 (d, *J* = 8.1 Hz, 1H), 7.74 (dd, *J* = 11.3, 4.1 Hz, 1H), 7.61 (t, *J* = 7.5 Hz, 1H), 7.47 (d, *J* = 9.0 Hz, 1H), 5.44–5.30 (m, 2H), 3.51 (dd, *J* = 13.1, 7.0 Hz, 2H), 2.02 (d, *J* = 4.7 Hz, 4H), 1.72–1.62 (m, 2H), 1.38–1.19 (m, 22H), 0.88 (t, *J* = 6.9 Hz, 3H). ^13^C-NMR (CDCl_3_): δ 161.73, 161.49, 154.73, 143.59, 135.60, 130.31, 129.88, 129.82, 129.08, 126.70, 121.96, 117.01, 116.26, 113.28, 40.00, 31.91, 29.78, 29.76, 29.70, 29.53, 29.45, 29.33, 29.26, 27.22, 27.07, 22.69, 14.13. ESI-HRMS (*m*/*z*): calcd. for C_32_H_43_NO_3_ [M − H]^−^: 488.31647; found: 488.31728.

*Methyl 3-oxo-3H-benzo[f]chromene-2-carboxylate* (**6a**). Yield: 92.1%; m.p.: 161.6–163.2 °C (Lit. 162–163 °C [32]). ^1^H-NMR (CDCl_3_): δ 9.39 (s, 1H), 8.35 (d, *J* = 8.3 Hz, 1H), 8.12 (d, *J* = 9.0 Hz, 1H), 7.94 (d, *J* = 8.1 Hz, 1H), 7.76 (m, 1H), 7.63 (m, 1H), 7.48 (d, *J* = 9.1 Hz, 1H), 4.02 (s, 3H). ^13^C-NMR (CDCl_3_): δ 164.24, 156.96, 156.12, 145.16, 136.36, 130.23, 129.45, 129.29, 129.21, 126.63, 121.53, 116.68, 116.03, 112.36, 53.05. ESI-HRMS (*m*/*z*): calcd. for C_15_H_10_O_4_ [M + Na]^+^: 277.04768; found: 277.04710.

*Ethyl 3-oxo-3H-benzo[f]chromene-2-carboxylate* (**6b**). Yield: 93.1%; m.p.: 116.6–118.1 °C (Lit. 118–119 °C [32]). ^1^H-NMR (CDCl_3_): δ 9.30 (s, 1H), 8.31 (d, *J* = 8.2 Hz, 1H), 8.10 (d, *J* = 9.0 Hz, 1H), 7.94 (d, *J* = 8.1 Hz, 1H), 7.76 (m, 1H), 7.63 (t, *J* = 7.5 Hz, 1H), 7.46 (m, 1H), 4.50 (d, *J* = 7.1 Hz, 2H), 1.48 (s, 3H). ^13^C-NMR (CDCl_3_): δ 163.56, 156.89, 155.94, 144.49, 136.14, 130.17, 129.38, 129.26, 129.13, 126.58, 121.47, 116.64, 116.40, 112.26, 62.09, 14.35. ESI-HRMS (*m*/*z*): calcd. for C_16_H_12_O_4_ [M + Na]^+^: 291.06333; found: 291.06174.

*n-Propyl 3-oxo-3H-benzo[f]chromene-2-carboxylate* (**6c**). Yield: 88.5%; m.p.: 85.2–86.9 °C (Lit. 86–88 °C [33]). ^1^H-NMR (CDCl_3_): δ 9.26 (s, 1H), 8.27 (d, *J* = 8.4 Hz, 1H), 8.07 (d, *J* = 9.0 Hz, 1H), 7.92 (d, *J* = 8.1 Hz, 1H), 7.74 (t, *J* = 7.7 Hz, 1H), 7.60 (t, *J* = 7.5 Hz, 1H), 7.43 (d, *J* = 9.0 Hz, 1H), 4.37 (t, *J* = 6.8 Hz, 2H), 1.86 (m, 2H), 1.08 (t, *J* = 7.4 Hz, 3H). ^13^C-NMR (CDCl_3_): δ 163.66, 156.83, 155.93, 144.37, 136.09, 130.15, 129.37, 129.26, 129.13, 126.57, 121.43, 116.64, 116.47, 112.24, 67.56, 22.07, 10.51. ESI-HRMS (*m*/*z*): calcd. for C_17_H_14_O_4_ [M + Na]^+^: 305.07898; found: 305.07756.

*Isopropyl 3-oxo-3H-benzo[f]chromene-2-carboxylate* (**6d**). Yield: 85.2%; m.p.: 95.2–97.9 °C. ^1^H-NMR (CDCl_3_): δ 9.23 (s, 1H), 8.29 (d, *J* = 8.4 Hz, 1H), 8.07 (d, *J* = 9.0 Hz, 1H), 7.92 (d, *J* = 8.1 Hz, 1H), 7.76–7.72 (m, 1H), 7.62–7.58 (m, 1H), 7.44 (d, *J* = 9.0 Hz, 1H), 5.32 (m, 1H), 1.44 (d, *J* = 6.3 Hz, 6H). ^13^C-NMR (CDCl_3_): δ 162.97, 156.88, 155.85, 143.96, 135.97, 130.17, 129.33, 129.26, 129.07, 126.54, 121.47, 116.89, 116.69, 112.25, 69.81, 29.72, 21.91. ESI-HRMS (*m*/*z*): calcd. for C_17_H_14_O_4_ [M + Na]^+^: 305.07898; found: 305.07824.

*n-Butyl 3-oxo-3H-benzo[f]chromene-2-carboxylate* (**6e**). Yield: 83.1%; m.p.: 60.1–62.6 °C (Lit. 60–62 °C [33]). ^1^H-NMR (CDCl_3_): δ 9.27 (s, 1H), 8.28 (d, *J* = 8.3 Hz, 1H), 8.08 (d, *J* = 9.0 Hz, 1H), 7.92 (d, *J* = 8.1 Hz, 1H), 7.74 (m, 1H), 7.60 (m, 1H), 7.44 (d, *J* = 9.0 Hz, 1H), 4.41 (t, *J* = 6.8 Hz, 2H), 1.94–1.77 (m, 2H), 1.54–1.47 (m, 2H), 1.03 (t, *J* = 7.4 Hz, 3H). ^13^C-NMR (CDCl_3_): δ 163.71, 156.86, 155.96, 144.42, 136.11, 130.18, 129.40, 129.27, 129.13, 126.57, 121.46, 116.68, 116.52, 112.28, 65.93, 30.70, 19.23, 13.82. ESI-HRMS (*m*/*z*): calcd. for C_18_H_16_O_4_ [M + Na]^+^: 319.09463; found: 319.09340.

*Tert-pentyl-3-oxo-3H-benzo[f]chromene-2-carboxylate* (**6f**). Yield: 83.6%; m.p.: 49.4–51.8 °C. ^1^H-NMR (CDCl_3_): δ 9.32 (s, 1H), 8.32 (d, *J* = 8.4 Hz, 1H), 8.11 (d, *J* = 9.0 Hz, 1H), 7.95 (d, *J* = 8.1 Hz, 1H), 7.77 (m, 1H), 7.66–7.61 (m, 1H), 7.48 (d, *J* = 9.0 Hz, 1H), 1.56–1.42 (m, 6H), 1.40 (m, 2H), 0.92 (t, *J* = 7.1 Hz, 3H). ^13^C-NMR (CDCl_3_): δ 164.35, 156.87, 151.96, 141.45, 136.11, 132.38, 129.21, 127.12, 121.34, 119.67, 117.18, 114.52, 107.49, 72.22, 36.81, 25.28, 8.73. ESI-HRMS (*m*/*z*): calcd. for C_19_H_18_O_4_ [M + Na]^+^: 333.11028; found: 333.11101.

*n-Hexyl 3-oxo-3H-benzo[f]chromene-2-carboxylate* (**6g**). Yield: 88.4%; m.p.: 44.3–46.7 °C. ^1^H-NMR (CDCl_3_): δ 9.09 (s, 1H), 8.15 (d, *J* = 8.5 Hz, 1H), 7.99 (d, *J* = 9.0 Hz, 1H), 7.85 (d, *J* = 8.1 Hz, 1H), 7.68 (t, *J* = 8.2 Hz, 1H), 7.58–7.53 (m, 1H), 7.35–7.30 (m, 1H), 4.37 (t, *J* = 6.9 Hz, 2H), 1.82–1.74 (m, 2H), 1.47–1.29 (m, 6H), 0.92–0.87 (m, 3H). ^13^C-NMR (CDCl_3_): δ 163.49, 156.67, 155.73, 144.07, 135.97, 130.00, 129.19, 129.09, 126.52, 121.27, 116.48, 116.26, 112.04, 66.13, 31.44, 28.59, 25.60, 22.55, 14.04. ESI-HRMS (*m*/*z*): calcd. for C_20_H_20_O_4_ [M + Na]^+^: 347.12593; found: 347.12554.

### 3.2. Biological Activity

#### 3.2.1. Cell Antiproliferative Activity Assay

The proliferation inhibitory activities of the synthesized target compounds **5a**–**i** and **6a**–**g** were evaluated using NSCLC cell lines (A549 and NCI-H460) by the MTT method *in vitro* with 7-HC and cisplatin as positive reference drugs. A549 and NCI-H460 cell lines were purchased from American Type Culture Collection (Rockville, MD, USA). Both cell lines were cultured in Dulbecco’s modified Eagle medium, 10% fetal bovine serum, 100 μg/mL penicillin, and 100 μg/mL streptomycin in humidified air at 37 °C. After the cells were seeded in 96-well culture plates and co-incubated with the compounds for different time, 20 μL of MTT solution (5 mg/mL) was added to each well and incubated for another 4 h at 37 °C. The formazan precipitate was dissolved in 100 μL DMSO and the absorbance at 495 nm of each well was measured using a microplate reader. All assays were conducted with four parallel samples. IC_50_ values were obtained by nonlinear regression using Origin software (version 8.0).

#### 3.2.2. Apoptosis Detection by Flow Cytometry

The extent of apoptosis was measured quantitatively by Annexin V binding assay [34]. Briefly, A549 cells in 60 mm dishes were treated with the indicated concentrations of compound **5e** or positive reference 7-HC at 37 °C. The cells floating in the supernatant were combined with the adherent fraction, washed with phosphate buffered saline (PBS) thrice, and incubated with Annexin V-FITC and PI for 15 min at 37 °C in the dark according to the manufacturer’s instructions (BD Biosciences, San Jose, CA, USA). The samples were immediately analyzed using a FACSCalibur flow cytometry system. The percentages of viable (Annexin V−/PI−), early apoptotic (Annexin V+/PI−), late apoptotic (Annexin V+/PI+), and necrotic (Annexin V−/PI+) cells were determined using FlowJo software (Tree Star, Inc., Ashland, OR, USA). For each sample, 1 × 10^4^ cells were collected. All experiments were performed in triplicate.

#### 3.2.3. Cell Cycle Distribution Analysis

Cell-cycle analysis was carried out by flow cytometry, as previously described [35]. In brief, after treatment with compound **5e** for different time, A549 cells were washed with PBS, fixed with 70% cold ethanol, and stored at −20 °C overnight. Fixed cells were washed with PBS again and sequentially incubated with RNase A (50 μg/mL) for 30 min and PI (50 μg/mL) for 15 min in the dark. The distribution of the cell cycle was determined by flow cytometry. The data were analyzed using ModFit software (Becton Dickinson, San Jose, CA, USA). All assays were conducted with three parallel samples, and 1 × 10^4^ cells were collected for each sample.

#### 3.2.4. Determination of Intracellular ROS Production

The DCFH-DA assay was used to determine the intracellular ROS level [36]. After treatment with compound **5e**, A549 cells were trypsinized, washed with PBS, and incubated with DCFH-DA at a final concentration of 10 μM for additional 20 min at 37 °C. The fluorescence intensity of the cell suspensions was measured using flow cytometry. For each sample, 1 × 10^4^ cells were collected. All experiments were performed in triplicate.

A fluorescence spectrometer was used for quantitative evaluation of intracellular ROS level changes [37]. After A549 cells were treated with compound **5e**, harvested and washed with PBS, the fluorescence intensity was detected using a Perkin Elmer LS55 spectrofluorimeter (PerkinElmer, Inc., Waltham, MA, USA). The results are expressed as the relative ROS level with the untreated cells. All experiments were performed in triplicate.

### 3.3. UV-Vis Absorption and Fluorescence Experiments

The UV-Vis spectra were recorded on an Agilent Cary 60 (Agilent Technologies, Santa Clara, CA, USA) spectrometer. The fluorescence emission was measured using the above spectrofluorimeter (see Section 3.2.4). The fluorescence quantum yield of QS in 0.1 M sulphuric acid solution was used as a reference standard. The following equation was used to calculate the fluorescence quantum yield of the target compounds:

(1) $$\text{Φs}=\text{Φr}\frac{I_{s}A_{r}n_{s}^{2}}{I_{r}A_{s}n_{r}^{2}}$$

where Φ is the fluorescence quantum yield. *I* stands for the integrated area under the emission curves. The subscripts s and r stand for sample and reference, respectively. *A* is the absorbance at a particular excitation wavelength; *n* is the refractive index of the medium. For living cell imaging, A549 cells were seeded on 35 mm glass-bottomed dishes at a density of 1 × 10^5^ cells/dish in culture medium and incubated overnight. The cells were treated with 10 and 20 μM of 7-HC, **5g**, and **6g**, respectively. After washing twice with PBS, the cells were immediately observed under a CLSM (Carl Zeiss LSM710, Oberkochen, Germany) using the excitation channel (λ_ex_ = 360 nm).

### 3.4. Data Analysis

Except where mentioned, data were presented as means ± SD. One-way analysis of variance test was performed on the data to assess the impact of the variables on the results (*n* = 3 or more). A *p* value of ≤0.05 was considered to be statistically significant.

## 4. Conclusions

In summary, two series of 3-oxo-3*H*-benzo[*f*]chromene-2-carboxylic acid derivatives were successfully prepared by a simple and convenient method. Among all the target compounds, **5e** showed the strongest proliferation inhibitory activity. *In vitro* pharmacological analysis demonstrated that compound **5e** exerted its activity against A549 cell line by inducing apoptosis and intracellular ROS generation, arresting cell cycle at the G0/G1 phase, suggesting it may be a promising lead for antitumor drug discovery in the future. Photophysical studies revealed that compound **6g** exhibited very low cytotoxicity and superior fluorescence properties, implying it may be a potential candidate for biological imaging.

## Supplementary Materials

Supplementary materials include the following items: (1) ^1^H-NMR spectra of the compounds **5e**, **5g**, and **6g**; (2) ^13^C-NMR spectrum of the compound **5g**; (3) HRMS spectra of the compounds **5e** and **6g**. These data can be accessed at: http://www.mdpi.com/1420-3049/20/10/18565/s1.
