# Supplementary material for: Synthesis, Fluorescence Properties, and Antiproliferative Potential of Several 3-Oxo-3H-benzo[f]chromene-2-carboxylic Acid Derivatives
Source: Molecules. 2015 Oct 13;20(10):18565–84. doi: 10.3390/molecules201018565 (PMC6332379; doi:10.3390/molecules201018565)
Supplement: Supplementary file 1 [file molecules-20-18565-s001.pdf]

Supplementary Materials

1. NMR and HRMS Spectra of Several Representative Target Compounds

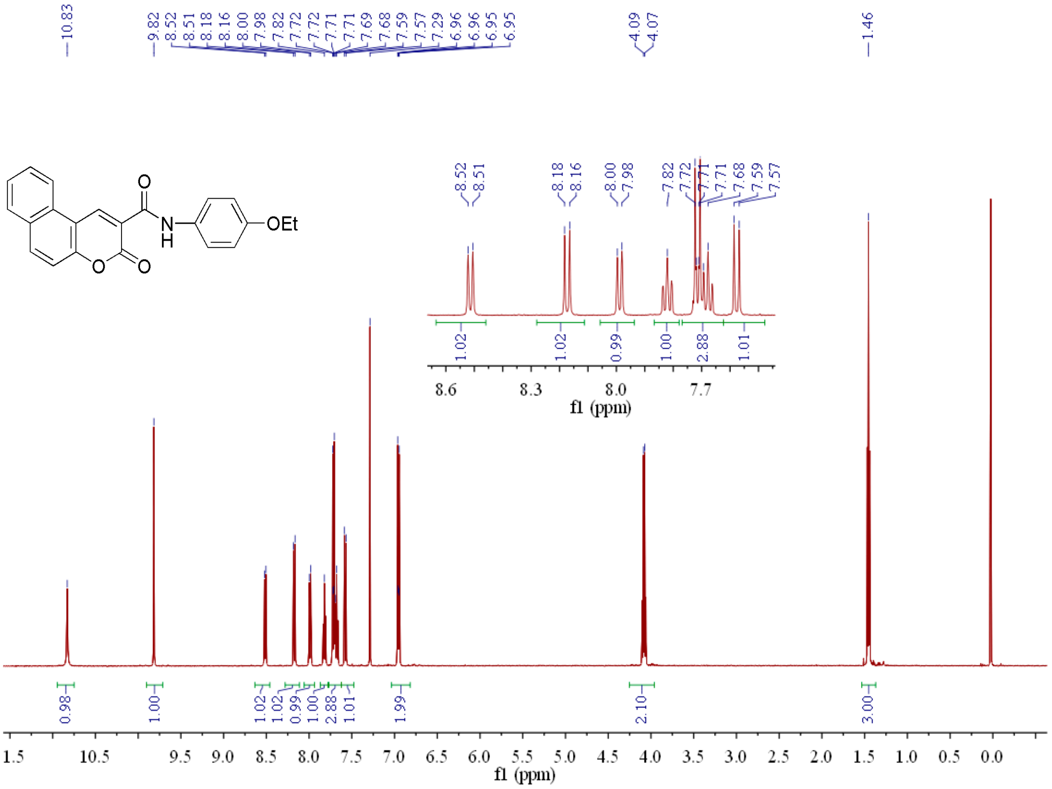

Figure S1. ^{1}H-NMR of *N*-(4-ethoxyphenyl)-3-oxo-3*H*-benzo[*f*]chromene-2-carboxamide (**5e**).

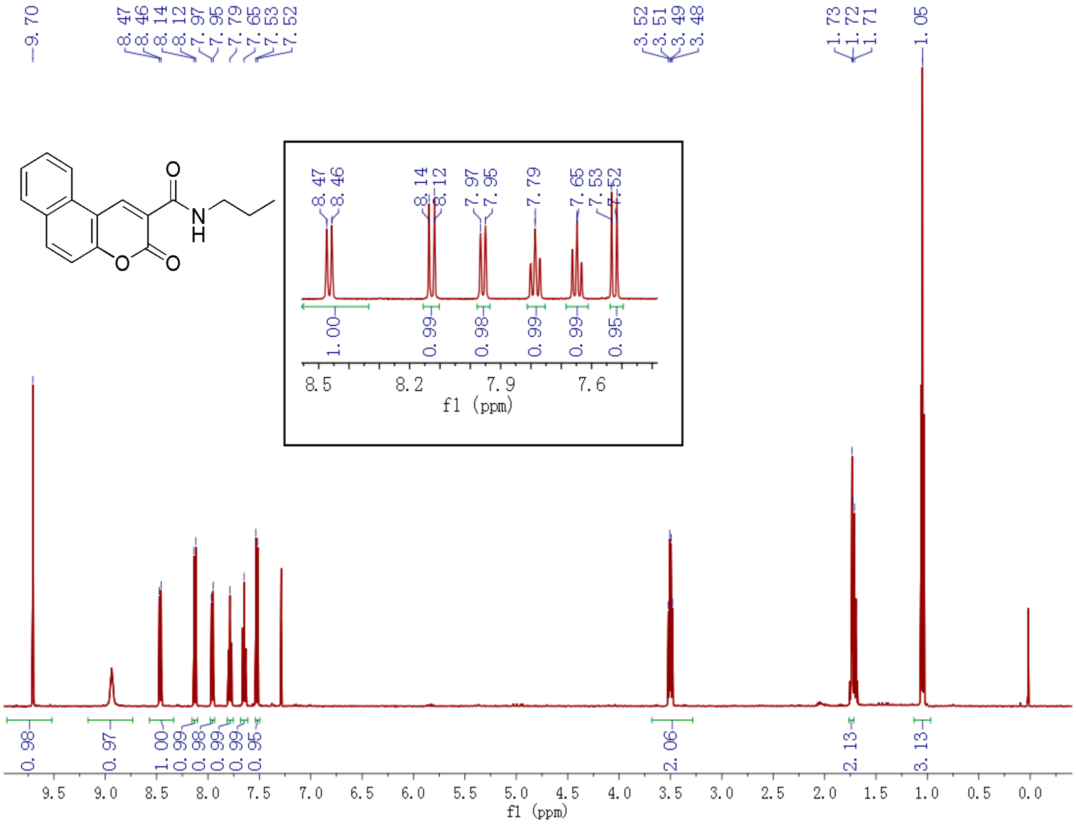

Figure S2. ^{1}H-NMR of 3-oxo-*N*-propyl-3*H*-benzo[*f*]chromene-2-carboxamide (**5g**).

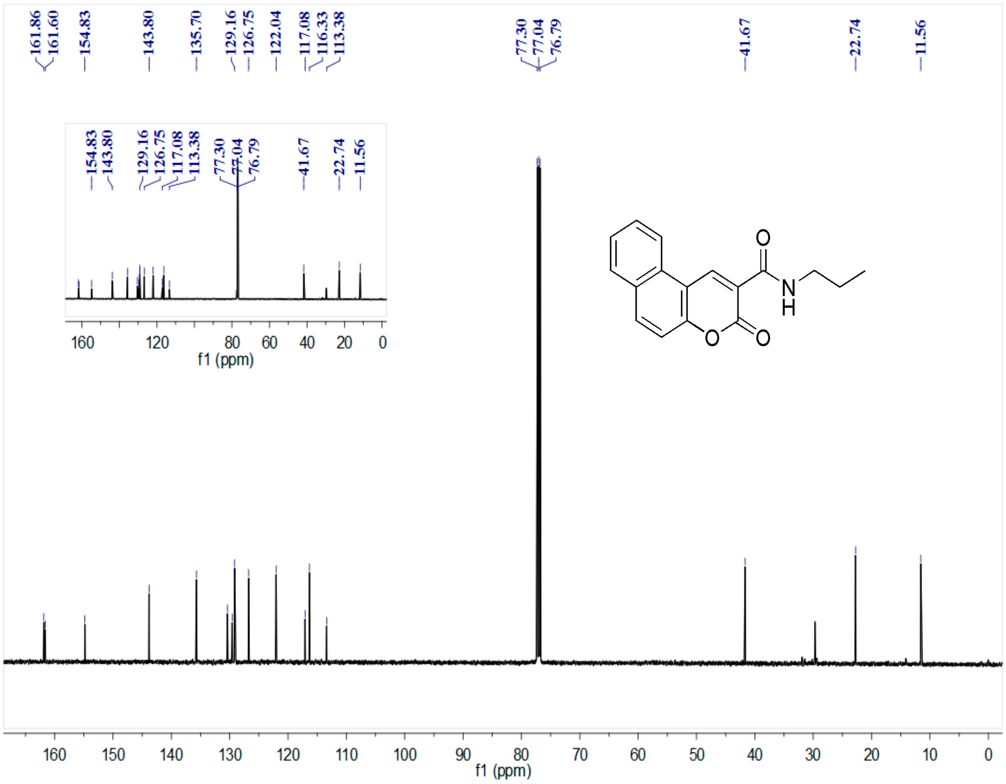

Figure S3. ^{13}C-NMR of 3-oxo-*N*-propyl-3*H*-benzo[*f*]chromene-2-carboxamide (**5g**).

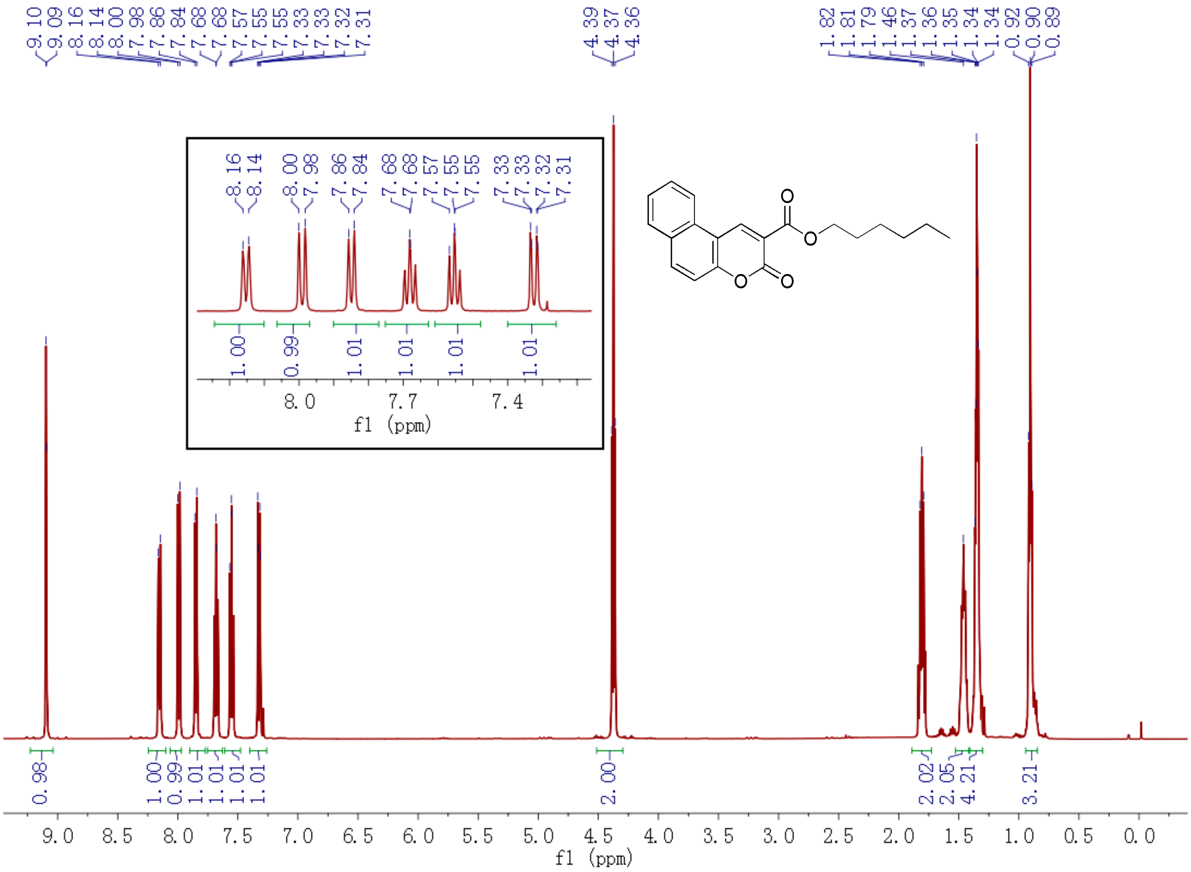

Figure S4. ^{1}H-NMR of *n*-hexyl 3-oxo-3*H*-benzo[*f*]chromene-2-carboxylate (**6g**).

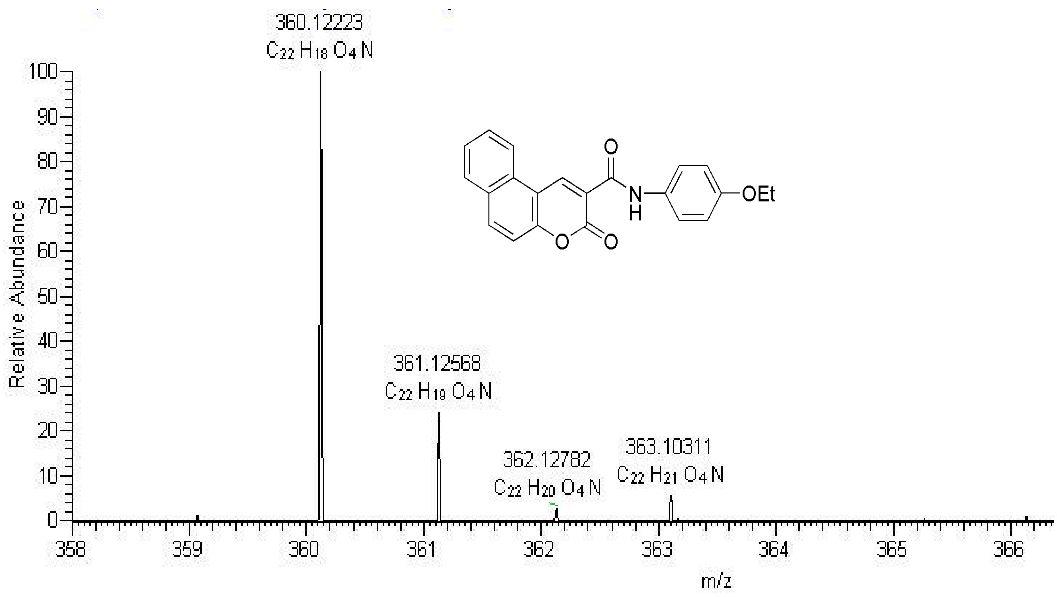

Figure S5. HRMS of *N*-(4-ethoxyphenyl)-3-oxo-3*H*-benzo[*f*]chromene-2-carboxamide (**5e**).

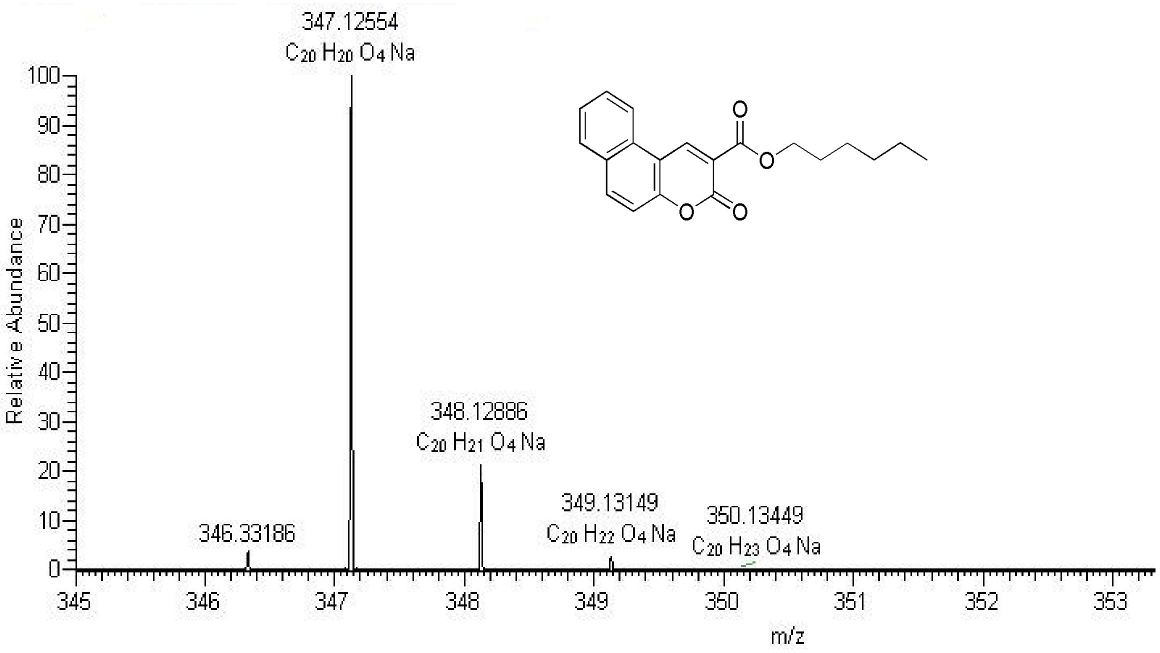

Figure S6. HRMS of *n*-hexyl 3-oxo-3*H*-benzo[*f*]chromene-2-carboxylate (**6g**).
